# Exploring older people’s understanding of the QOL-ACC, a new preference-based quality-of-life measure, for quality assessment and economic evaluation in aged care: the impact of cognitive impairment and dementia

**DOI:** 10.1186/s12955-023-02222-x

**Published:** 2024-01-07

**Authors:** Kiri Lay, Matthew Crocker, Lidia Engel, Julie Ratcliffe, Rachel Milte, Claire Hutchinson

**Affiliations:** 1 Health and Social Care Economics Group, Caring Futures Institute, Flinders University, GPO Box 2100, Adelaide, Adelaide, SA 5001 Australia; 2 Health Economics Division, School of Public Health and Preventive Medicine, Monash University, Melbourne, VIC Australia

**Keywords:** Aged care, Think-aloud, Cognitive impairment, Self-report

## Abstract

**Background:**

Quality-of-life is an essential outcome for quality assessment and economic evaluation in health and social care. The-Quality-of-Life – Aged Care Consumers (QOL-ACC) is a new preference-based quality-of-life measure, psychometrically validated with older people in aged care. More evidence is needed to inform the self-report reliability of the QOL-ACC in older people with varying levels of cognitive impairment and dementia.

**Methods:**

A think-aloud protocol was developed and applied with older residents. The Mini Mental State Examination (MMSE) was applied to assign participants to no cognitive impairment (NCI - MMSE score ≥ 27) and cognitive impairment (MMCI - MMSE score < 27) subgroups. Three independent raters utilised a Tourangeau survey response model-based framework to identify response issues. Data were compared across cognition subgroups and synthesized using a ‘traffic light’ grading to classify frequency and type of response issues. Gradings were utilised to assess self-report reliability according to different levels of cognitive impairment.

**Results:**

Qualitative data from 44 participants (NCI = 20, MMCI = 24) were included for analysis. Response issues were more evident in the cognitive impairment subgroup than the no cognitive impairment subgroup. All participants who received a ‘red’ grade had an MMSE score of < 20 and 66% of ‘amber’ grades occurred in the cognitive impairment subgroup.

**Conclusions:**

The QOL-ACC is able to be completed reliably by older residents with an MMSE score > 17. Future research is needed to assess the generalisability of these findings to other preference-based quality of life instruments and for older people in other care settings including health systems.

**Supplementary Information:**

The online version contains supplementary material available at 10.1186/s12955-023-02222-x.

## Background

Quality of life is the most important outcome for economic evaluation and an important person-centred quality assessment indicator; with commonly applied measures capturing either generic or condition specific quality of life [1, 2]. A key tenet of all measures is the reporting of outcomes from the perspective of the person themselves, and as such self-report is preferred over proxy completion [3, 4]. However, in populations of older people (aged 65 years and over) in aged care settings where cognitive impairment and dementia are prevalent, proxy completion is often the default [5]. This is despite a significant body of research indicating that proxy completion is not equivalent to self-report due to low levels of proxy-person agreement [6]. Given that in Australia and the United States over 50% of older people living in residential care facilities have a diagnosis of dementia [7] with this percentage even higher in the United Kingdom (70%) [8], evidence as to what extent older people with cognitive impairment and dementia can reliably self-report their quality of life using standardised self-report measures is especially important for the measurement and valuation of quality of life in an aged care population.

In Australia, the Quality of Life – Aged Care Consumers (QOL-ACC) tool has been recently adopted by the Department of Health and Aged Care to measure quality of life in the expanded Mandatory National Quality Indicators Program for residential aged care, introduced in April 2023 [9]. The QOL-ACC is a new preference-based quality of life measure developed from its inception with older people accessing aged care services. The instrument has been comprehensively psychometrically tested and validated with older people receiving aged care services, including older people with mild cognitive impairment [10, 11]. However, there is a need for more detailed evidence on the ability of older people with cognitive impairment and dementia to reliably self-report quality of life.

This study applied a qualitative ‘think-aloud’ approach with a sample of aged care residents with varying levels of cognition, firstly to examine how older aged care residents’ understand and respond to the QOL-ACC and, secondly, to examine the reliability of older aged care residents’ self-report using the QOL-ACC tool. To address the first objective, a think-aloud approach was adopted and data analysed for response issues utilising a Tourangeau four-stage response model based framework [12]. To address the second objective, a ‘traffic light’ approach was used to synthesise think aloud findings and provide guidance to aged care providers and researchers on the level of cognition beyond which proxy-report may be needed to compliment self-report.

## Methods

### Participants

Aged care residents were recruited from 10 facilities across urban and rural South Australia. Residential care facilities, in Australia, provide care primarily for older people requiring a level of care incompatible with independent living. Care provided in residential facilities can include one, or a combination of support with activities of daily living (e.g. personal care, meals, laundry); cognition and behaviour support and complex health care. In Australia residential aged care places are funded based on a means-tested co-contribution system with a mix of user-pays and Government subsidies and supplements paid directly to the facility [13]. Residents below 65 years of age and those with a Mini Mental State Examination (MMSE) score of 10 or below (indicating the presence of severe cognitive impairment or dementia) were excluded. Residents were included if they were permanent residents at an aged care facility (i.e. not in respite care) and could speak and understand English. Residents were purposively sampled into two subgroups, those with no cognitive impairment and those with mild to moderate cognitive impairment. Groupings were based on MMSE scoring guidelines identified by the UK’s National Institute of Care Excellence, where an MMSE score of ≥ 27 signifies no cognitive impairment (NCI), 21–26 equals mild cognitive impairment and 10–20 equals moderate cognitive impairment [14]. A target of 20 participants in each cognition sub-group was identified based on guidelines for think-aloud studies suggesting a target sample of 15 is sufficient to achieve saturation [15].

### Materials

Cognition was assessed using the MMSE, a validated measure of cognitive capacity and the most widely used cognition assessment tool internationally [16]. Researchers were trained in the administration of the MMSE prior to data collection [17]. Key socio-demographic data were collected including age and time in residential care as well as level of education and country of birth.

Quality of life was assessed using the QOL-ACC, a preference-based measure of quality of life developed with and for aged care recipients. An aged care user specific preference-based scoring algorithm is available to allow the conversion of individual responses to the QOL-ACC descriptive system into utilities on the Quality Adjusted Life Year (QALY) scale for the purposes of economic evaluation [18]. The QOL-ACC consists of 6 dimensions; *Mobility, Pain Management, Emotional Well-being, Social Relationships, Independence and Activities*. The QOL-ACC has a five-level response scale for each dimension ranging from ‘All of the time’ to ‘None of the time’ and a recall period of ‘today’ [19].

### Procedure

Eligible residents who met the study inclusion criteria and consented to participate undertook a practice think-aloud task Following the practice, a paper copy of the QOL-ACC self-report version was given to the resident, and they were reminded again to think-aloud while completing the questions (concurrent think aloud). If the resident was silent for one question they were stopped and asked to verbalise their thought processes for the previous question using semi-scripted verbal probes (retrospective think aloud). The think-aloud section of the interview was audio recorded.

### Analysis

Audio recordings were transcribed and coded in NVIVO qualitative analysis software for text relating to each dimension. This text was then anonymised and exported into a Microsoft excel spreadsheet. Three coders independently coded the text for response issues using a framework developed from Tourangeau’s four stage response model [12]. Tourangeau’s model comprises four response stages, comprehension, recall, judgement and response mapping, where comprehension refers to the understanding of the domain descriptor, recall refers to the ability to recall appropriate information and adhere to the correct recall period (‘today’), judgement is described as the ability to assess the information and formulate an appropriate response, and response mapping involves mapping verbal response to the available response options. An additional ‘struggle’ category was included in the analysis based on work by Horwood [20] and Al-Janabi [21] to capture instances where participants required interviewer assistance, for example, redirection or reminders, in order to complete the survey task. For a more comprehensive description see additional file (Additional File 1). Interrater reliability was estimated using percentage agreement and Gwet’s AC1 [22], after which the three coders met to discuss and resolve conflicts. Detailed notes were made on the coders’ interpretation of the source of each recorded response issue. Coders were blinded to demographic details of participants’, including MMSE scores.

Descriptive statistics were generated for socio-demographic factors and Fisher’s Exact test [23]was used to test for between group differences. Response issues were counted and totalled for each individual participant overall as well as for each response stage and dimension. Fisher’s exact test was also used to test for difference in response issue frequency overall and for each response stage by MMSE sub-group, education, age, and gender. Spearman’s Rho was used to test for correlation between raw MMSE score and response issue frequency overall. QOL-ACC utility scores were computed using the main aged care user (Australian older adult population) preference-based scoring algorithm [18].

### Traffic light

To synthesise response issue data and assess how cognition level affected the reliability of participants’ self-report, each participant was assigned a ‘traffic light’ grade based on their response issue frequency and type. The traffic light grades reflected the extent of the reliability of self-report where, a ‘red’ grade indicated that there was sufficient evidence to suggest that the participant was not able to reliably self-report, an ‘amber’ grade reflected inconsistent or inconclusive evidence as to the reliability of the participants’ self-report and ‘green’ indicated the participant was able to reliably self-report. Participants were graded ‘red’ if they experienced a ‘struggle’ type response issue as well as > 1 additional response issues. Participants who experienced no more than 1 issue and no ‘struggle’ type issues were awarded a ‘green’ grade. Those who experienced > 1 response issue but no struggle were awarded an ‘amber’ grade. The grading guide is summarised in Table 1. These grades were then mapped against MMSE scores and visually inspected to identify patterns in traffic light grade by MMSE score and cognition subgroup.

**Table 1:** Traffic light grading description

Traffic light grade	Grading criteria
GREEN	≤ 1 response issue and no ‘struggle’ type issue.
AMBER	> 1 response issue and no ‘struggle’ type issue.
RED	> 1 response issue plus a ‘struggle type issue.

## Results

### Participants

In total, 46 residents provided full consent and were interviewed across 11 facilities. Two participants received MMSE scores below the cut-off of 10 (indicating the presence of severe dementia) and, whilst they participated in the interview, their data were excluded. The resulting 44 total participants, n = 24 in the no cognitive impairment (NCI) sub-group and n = 20 in the moderate or mild cognitive impairment (MMCI) sub-group exceeded the target of 20 sample size of 20 participants per subgroup. Over 60% of participants were female with a higher proportion of female to male in the MMCI sub-group compared with the NCI sub-group. The MMCI sub-group was also slightly older with a mean age of 88.2 compared with the NCI mean age of 85.6. Almost all participants, across both sub-groups were born in Australia (90.9%) and most (84.1%) were living in regional facilities. Mean QOL-ACC utility scores were higher for the MMCI sub-group (m = 0.771) than the NCI group (m = 0.751). Higher QOL-ACC utility scores indicate higher overall Quality of Life. Fisher’s exact tests showed no statistically significant between group differences on most socio-demographic factors. A Significant association was found between education level and cognitive impairment sub-group (p = .025). Demographic information is detailed in Table 2.

**Table 2:** Demographic information

Cognition Group (MMSE)			
	No Cognitive Impairment	Mild/Moderate Cognitive Impairment	Total
TOTAL	24	20	44
Age:			
Mean (SD)	85.6 (8.58)	88.2 (6.75)	87.05 (7.72)
Median (25th & 75th percentiles)	86 (79, 93)	88 (83, 92)	86.5 (81.25, 92.75)
Gender: n (%)			
Female	13 (54)	15 (75)	28 (64)
Male	11 (46)	5 (25)	16 (36)
Education: n (%)*			
Primary school	2 (8)	7 (35)	9 (20)
Some secondary school	13 (54)	12 (60)	25 (57)
Completed secondary school	4 (17)	0 (0.00)	4 (9)
Tertiary (vocational or university)	5 (21)	1 (5)	6 (14)
Living resi. care: n (%)			
< 12 m	4 (17)	5 (25)	9 (20)
1-3y	10 (42)	7 (35)	17 (39)
≥ 3y	9 (37)	6 (30)	15 (34)
Missing	1 (4)	2 (10)	3 (7)
Birth country: n (%)			
Australia	23 (96)	17 (85)	40 (91)
England	0 (0)	2 (10)	2 (4.5)
Other	1 (4)	1 (5)	2 (4.5)
Location: n (%)			
Metropolitan	4 (17)	3 (15)	7 (15.90)
Regional	20 (83)	17 (85)	37 (84.10)
QOL-ACC Utility score:			
Mean (SD)	0.751 (0.243)	0.771 (0.270)	0.758 (0.253)
Median (25th & 75th percentiles)	0.805 (0.640, 0.945)	0.868 (0.717, 0.927)	0.813 (0.647, 0.932)

* *P* = > 0.05

Interrater agreement calculated with Gwet’s AC1 was 0.74, 95%CI (0.69,0.78) and percentage agreement was 75%, representing good agreement. All conflicts were resolved through the group discussion process. A table with example quotes of responses coded ‘response issues’ and ‘no response issues’ is included in online supplementary information 1.

### Response issues by response stage

Issues were identified for both cognition subgroups across all dimensions. Table 3 presents the total response issues identified for both cognition sub-groups, by response stage and dimension. The total number of participants is also shown as it is possible for more than one issue to be identified for each participant. When examining the percentage of participants in each sub-group experiencing issues the evidence is mixed, with more participants in the NCI group experiencing issues with the ‘Pain Management’ and ‘Activities’ dimensions. However, only participants in MMCI sub-group had more than one issue identified for any one response (n = 3). The ‘Mobility’ dimension had the highest number of response issues overall (n = 15) with most of these occurring in the comprehension response stage. The struggle issue type mostly occurred with the ‘Mobility’ and ‘Pain Management’ dimensions (n = 6 of 8). No participants in the NCI sub-group experienced a struggle type issue whereas 8 participants in the CI group did. This was a statistically significant difference (p = .008). Comprehension issues were also more prevalent in the MMCI sub-group however this difference did not reach statistical significance.

**Table 3:** Number of response issues by dimension and cognition subgroup

	Mobility			Pain Management		Emotional Well-being		Social Relationships		Independence		Activities		
		NCI	MMCI	NCI	MMCI	NCI	MMCI	NCI	MMCI	NCI	MMCI	NCI	MMCI	
Comprehension		4	5	1	2	0	0	0	0	1	3	0	0	
Recall		0	0	0	0	3	1	0	0	0	2	0	0	
Judgement		0	0	0	0	0	0	0	0	0	0	1	0	
Response Mapping		2	1	3	3	1	0	1	3	0	1	1	3	
Struggle		0	3	0	3	0	1	0	1	0	0	0	0	
**Total**		**6**	**9**	**4**	**8**	**4**	**2**	**1**	**4**	**1**	**6**	**2**	**3**	
N Participants who had issues (%)		5 (21)	8(40)	5 (21)	6 (30)	4(17)	1(5)	1 (4)	4 (19)	1 (4)	6 (30)	3 (13)	2 (10)	

### Response issues overall

**Fig. 1:**
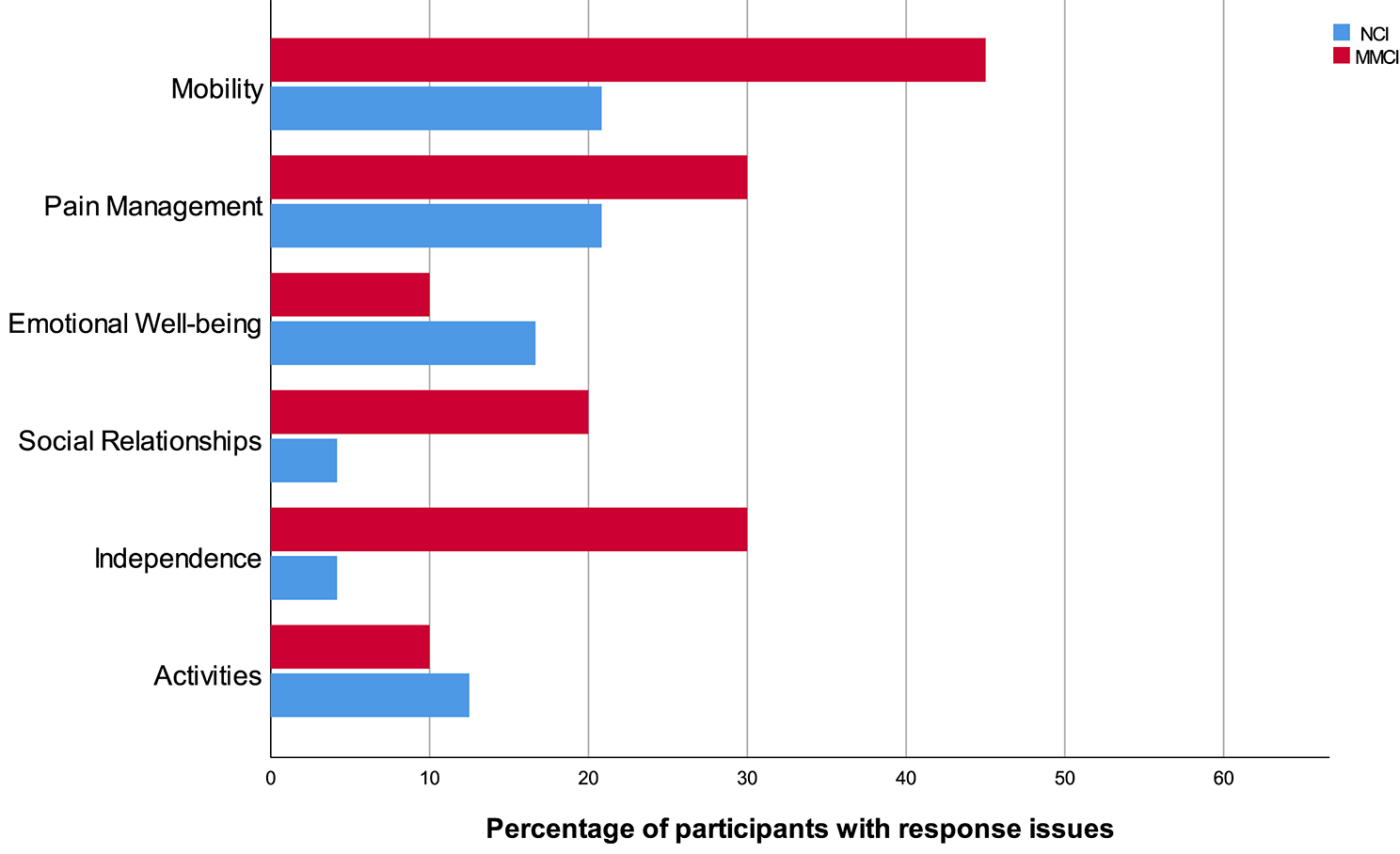
Percentage of participants with response issues by dimension and cognition subgroup

Across all dimensions and response stages, more issues were identified for the MMCI group (n = 32 issues total) than for the NCI group (n = 18 issues total). Relatively more participants from the MMCI sub-group experienced multiple response issues (> 1) across all dimensions (n = 9, 40.9%), than those in the NCI group (n = 2, 8.7%). This difference reached statistical significance (p = .045). Fisher’s exact test also revealed a statistically significant difference in participants experiencing any errors by cognition subgroup, (p = .036). Figure 1. shows the percentage of participants who experienced issues on each dimension by cognition sub-group. A higher percentage of participants in the MMCI sub-group experienced response issues for each dimension with the exception of *Activities* and *Emotional well-being*: Spearman’s rank correlation was computed and showed MMSE score was negatively correlated with the total number of response issues, r(43) = − 0.41, p = .005. Fisher’s exact tests revealed no statistically significant differences in issue frequency by education, age-group, gender or time in facility.

### Traffic light grade

**Fig. 2:**
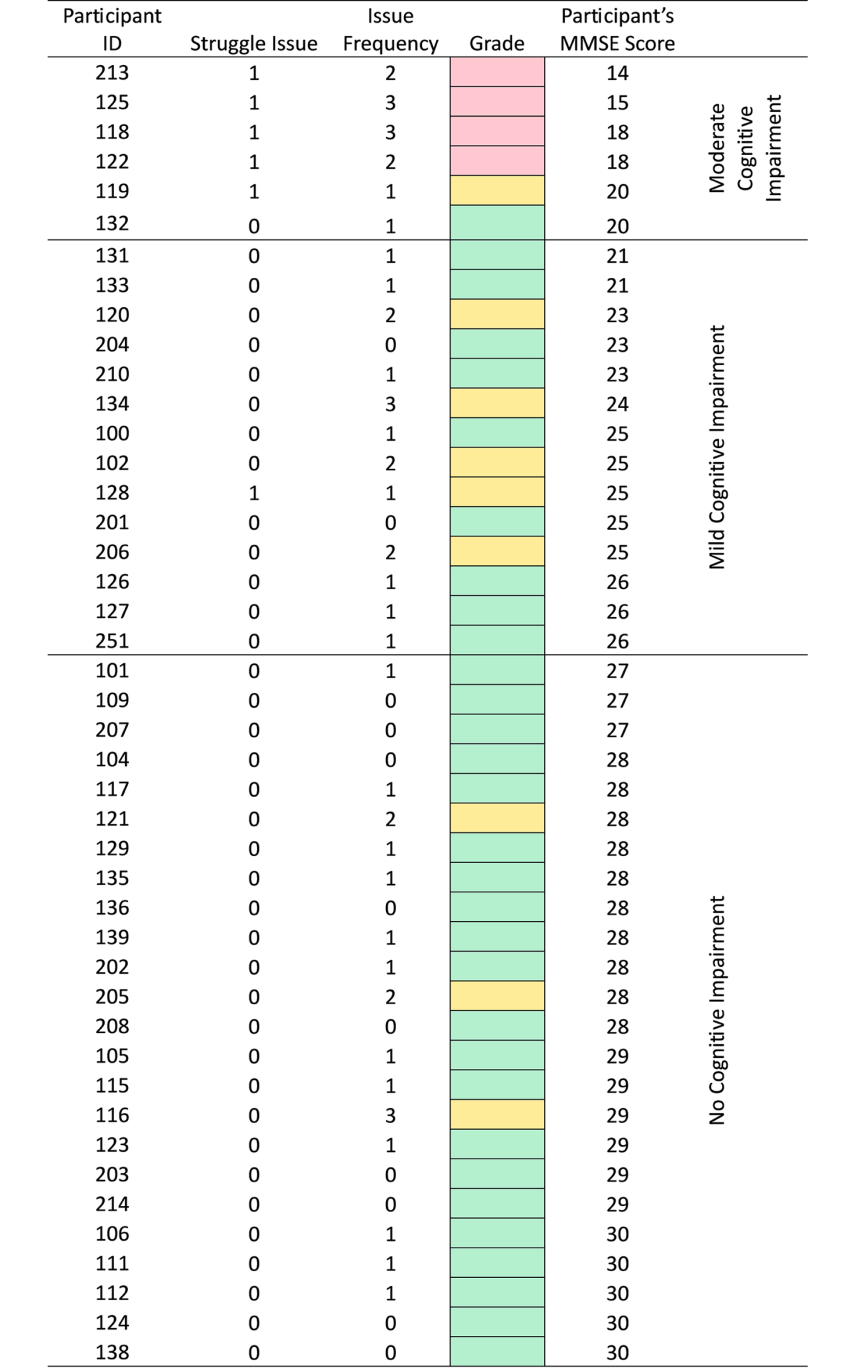
Participant level issues and traffic light grade – ordered by MMSE score

A relatively small number of red grades were assigned to participants overall (n = 4) and all were assigned to participants with an MMSE score of < 20. There were nine participants assigned amber grades, with two thirds of these assigned to participants in the MMCI sub-group. Of the 22 participants in the NCI subgroup, n = 3 were assigned an amber grade. One of the six participants with an MMSE Score indicating moderate cognitive impairment, was assigned a green grade. Participant level traffic light grading results, ordered by MMSE score are presented in Fig. 2.

### Qualitative analysis

#### Mobility

The wording of the domain descriptor for ‘getting around’ allows for a report of full mobility even if the participant uses aids to navigate outside and inside areas. Some participants however reported their frequency of mobility aid use rather than their ability/inability to get around. The example quote below illustrates this issue which was coded as a comprehension issue.

*I don’t get on with a stick because it trips me. I use my wheelchair now and again, but very seldom. Most of the time, some of the time, no, most of the time. All of the time I use the walker. All of the time, yeah.* (Participant 10, MMCI group – Participant selected ‘All of the time’)

This was the primary response issue for this dimension for both cognition subgroups (MMCI = 3, NCI = 4). There was also a relatively high prevalence of ‘struggle’ type issues on this dimension (n = 3) as participants experienced difficulties in understanding or completing the task including, attempting to indicate a positive response to all five response options (as opposed to choosing the single response option which most reflected their own mobility level), moving onto subsequent questions before selecting a response and becoming side-tracked and forgetting about the survey task.

#### Pain management

The leading response issue encountered by participants within the *Pain management* dimension was an attempt to answer the question accounting for pain severity rather than the efficacy of pain management (n = 7 participants). Whilst pain severity can be impacted by the management (or otherwise) of pain symptoms, this was coded as a comprehension issue where participants explicitly considered pain severity unrelated to pain treatment as in the below example.

*The pain is only when I’m standing on my legs, well, all of the time I can. I could say all of the time, I suppose, because I’m sitting down quite comfortably….I can sit down and lay down, no pain at all. Just that when I’m standing it varies sometimes, especially with all this. I’ll put most of the time, I think.* (Participant 111, NCI group – Participant selected ‘most of the time.)

A related issue experienced by a small number of participants from both cognition sub-groups (N = 3) was how to answer where they reportedly did not experience pain at all as illustrated with the below quote. This response was coded as a response mapping issue.

*When I experience pain, it is well managed. I suppose they don’t give me anything for the pain so I don’t have any pain so I guess that’s none of the time. Does that make sense? Oh, what I understand by it. Wait a minute. Perhaps I’d better put all of the - there’s something wrong with that question. I’m going to put there, N/A, am I allowed to do that?* (Participant 105, NCI group – Participant selected ‘All of the time’)

#### Emotional well-being

For the Emotional well-being dimension, some participants struggled with the ‘today’ timeframe and responded by averaging over longer time periods (n = 4). These participants framed their answers with reference to their general personal characteristics rather than reference to their current situation. This response, illustrated with the following quote, was coded as a ‘recall’ issue.

*I’m generally happy. Well, sometimes I’m happy. Not happy all the time though. I don’t think anyone’s happy all the time, are they? Definitely not all the time. I’d say on the weekend I’ll be happy because I’ll be with my family. So, I’ll put some of the time.* (Participant 139, NCI group – Participant selected ‘Some of the time’)

#### Social relationships

There were two instances of participants selecting a response that differed from the response they indicated they intended to select for the *Social Relationships* dimension e.g. expressing that they had good social relationships with friends and family but then indicating a response which indicated otherwise. Other than this, there were no identified issues for this dimension that were experienced by more than one participant. One participant expressed dissatisfaction with the combination of ‘family’ and ‘friends’ in the question wording. This participant believed that their response would differ between these two groups and they felt forced to average. Another participant had an issue defining the difference between ‘some of the time’ and ‘all of the time’. These issues were all coded as Response Mapping issues.

*I’ve always had a good relationship with my family. Friends, I’m afraid, are – there aren’t too many around now. Yes. I have a good relationship with my family. I don’t – to be quite honest, I don’t get many friends here. Not because I’m unsocial but because most of my friends have passed away, so just a little of the time. But that doesn’t include my family, of course. Well, family, yes, all of the time.* (Participant 102, MMCI group – Participant selected ‘All of the time’)

#### Independence

Independence was interpreted by some as related to their ability to move around independently (mobility) rather than their ability to make choices for their own lives. This issue was coded as a comprehension issue and primarily experienced by participants in the cognitive impairment sub-group (NCI = 4, MMCI = 1).

*I’ll put most of the time there, too, I think….Because I have to wear stretch stockings because I’ve got varicose veins, and that holds me up a little bit. It takes a little while to get mobile and then I’m right.* (Participant 209, MMCI group – Participant selected ‘Most of the time’)

#### Activities

For the *activities* dimension, as compared to the previous five dimensions, there were a higher number of participants who selected an answer that was inconsistent with their verbal data (n = 4;); this was coded as a response mapping issue. An example of this issue is below.

Participant: *Yeah. Most of the time.*

Interviewer: *And what are some things that you’re thinking about?*

Participant: *Reading*. (Participant 121, NCI group – participant selected ‘All of the time’)

Though this issue was more prevalent in this dimension and in the NCI group, it was not specific to this dimension occurring 11 times across all dimensions and experienced by participants from both cognition sub-groups.

## Discussion

This study applied a qualitative think-aloud approach to identify response issues and generate evidence to inform guidance as to the level of cognition beyond which proxy completion of the QOL-ACC may be preferred over self-report. Relative to older people without cognitive impairment, the cognitive impairment subgroup experienced more response issues overall and participants in the cognitive impairment subgroup were more likely to experience multiple response issues. Based on the comparison of traffic light grades and MMSE scores, the findings from this study indicate that older aged care residents with an MMSE score of 20 and above can reliably self-report their QOL using the QOL-ACC tool. No participants in our sample had an MMSE score of 19, and potentially therefore this score may also be within the range for reliable self-report.

A key issue for older people in both the NCI and MMCI subgroups was the lack of adherence to the required recall period of ‘today’. This was more prevalent for the ‘happy’ and ‘pain management’ dimensions as participants attempted to provide a meaningful response, incorporating fluctuating states. This issue has been identified with several condition specific and generic quality of life measures in both health [24, 25] and aged care settings [26, 27]. It is an ongoing challenge to select a recall period that is recent and specific enough to enable the recollection and assessment of relevant information and broad enough incorporate fluctuations but not so broad that it fails to capture changing health states [28, 29].

Struggle type issues occurred more frequently at the beginning of the survey task, with none recorded for the final two dimensions. This may have been due to participants becoming familiar with the question and response format. Whilst all participants had completed a ‘warm-up’ task with the same question and response format, this was not sufficient to prevent these struggle type issues for the participants with the lowest MMSE scores. With the addition of an interviewer to guide respondents through the survey task, more older adults with moderate cognitive impairment may be able to reliably self-report.

No current guidelines exist for when proxy report should be preferred for most generic preference-based measures of QoL. Analysis of the validity of self-report QoL measures in older cognitive impaired populations using psychometric methods reveal divergent findings. For example, a recent systematic review of the psychometric performance of the EQ-5D-5 L in people with dementia found evidence of acceptable convergent validity and known group validity, however there was no consistent evidence for responsiveness. Additionally, only half of studies found the EQ-5D-5 L to be acceptable based on missing data (n = 6) or a subjective measure of participants ‘ability to complete’ the measure [30]. Given the mixed psychometric some have highlighted the need for more qualitative research with these populations to identify the content validity of QoL tools [31]. A recent qualitative study by Ratcliffe and colleagues to determine self-report reliability for the EQ-5D-5 L, in older aged care residents with cognitive impairment, indicated that an MMSE score of ≥ 23, (representing no cognitive impairment and the upper levels of mild cognitive impairment) is appropriate for reliable self-report of the EQ-5D-5 L [32].

Guidelines for when proxy report should be preferred over self-report are available for some dementia-specific QOL instruments [33], with some reportedly able to be completed by people with MMSE scores as low as 10, albeit with interviewer assistance [34–36]. Comprehension of these instruments is often facilitated by easy-read or pictorial adjustments and interviewer administration [37]. Interviewer administration may not always be universally possible in larger populations due to practical and resource limitations.

This study has several limitations which are important to highlight. Firstly, some participants were reluctant or unable to verbalise their thoughts, despite interviewer prompting, leading to limited think-aloud data for these participants, potentially impacting on the identification of response issues. Participants may also have been experiencing a higher level of cognitive fatigue that they would under normal conditions, potentially leading to more response errors overall. Response mapping issues, primarily where the selected response contradicted the verbally expressed intended response, were more common for the final two dimensions, indicating these response issues could potentially have been caused by respondent fatigue. The interviewer was new to the resident and a considerable process of participant consent, MMSE and demographic questions had been undertaken prior to the completion of the QOL-ACC. Additionally the added cognitive burden of the think-aloud task may have exacerbated fatigue for participants, leading to an increase in this type of response issue. Finally, the reliability of the statistically significant results found in this study is limited by the relatively small sample size for quantitative analysis.

The MMSE is the most widely used cognitive assessment tool however it has been found to have limitations in the areas of verbal fluency and reasoning/judgement [16, 38]. It may be that different or further measures of cognitive impairment may provide a fuller picture of the aspects of cognition which impact on ability to reliably self-report. Additionally, other standardised dementia screening tools are commonly utilised in aged care settings with no single standardised tool utilised for pre-residential aged care assessments in Australia. This lack of consistency could have practical implications for developing guidelines for self vs. proxy completion.

For large-scale quality assessment exercises, using a standardised measure of cognition is likely to be the most practical approach to determine residents’ ability to self-report. Though facilities may not have the resources to perform cognition assessments concurrently with quality assessments, the results of this study indicate that aged care residents with mild cognitive impairment and those in the upper bands of moderate cognitive impairment, can complete the QOL-ACC reliably and without assistance.

## Conclusions

The findings from this novel exploratory study indicate that the QOL-ACC survey tool is generally well understood and able to be reliably and independently self-reported by older aged-care residents with an MMSE score between 18 and 30. Considering the study limitations previously highlighted, caution should be taken in assuming that residents with an MMSE scores < 18 cannot reliably self-report their QOL using the QOL-ACC tool. Additionally residents with MMSE scores below 18 may be able to complete the QOL-ACC reliably with the assistance of an interviewer. Future research should focus on interviews with a sample of older people with moderate cognitive impairment (concentrated in the 10–20 MMSE range) to provide more detailed evidence on reliable self-report in this population.

### Electronic supplementary material

Below is the link to the electronic supplementary material.

Supplementary Material 1

## Data Availability

The datasets generated during the current study are not publicly available due to Flinders University ethics requirements but are available from the corresponding author on reasonable request.

## Abbreviations

QOL-ACC: Quality of Life Aged Care Consumers
EQ-5D-5L: EuroQoL – Five dimensions – Five Levels
MMSE: Mini Mental State Examination
CI: Confidence Interval
NCI: No Cognitive Impairment
MMCI: Mild/Moderate Cognitive Impairment
QALY: Quality Adjusted Life Year
