# Supplementary material for: Exploring older people’s understanding of the QOL-ACC, a new preference-based quality-of-life measure, for quality assessment and economic evaluation in aged care: the impact of cognitive impairment and dementia
Source: Health Qual Life Outcomes. 2024 Jan 7;22:4. doi: 10.1186/s12955-023-02222-x (PMC10773014; doi:10.1186/s12955-023-02222-x)
Supplement: Supplementary file 1 — Supplementary Material 1 [file 12955_2023_2222_MOESM1_ESM.docx]

Online Supplementary Information 1.

Exploring older people’s understanding of the QOL-ACC, a new preference-based quality-of-life measure, for quality assessment and economic evaluation in aged care: the impact of cognitive impairment and dementia.

*Health and Quality of Life Outcomes*

Kiri Lay^1^, Matthew Crocker^1^, Lidia Engel^2^, Julie Ratcliffe^1^, Rachel Milte^1^, Claire Hutchinson^1^

^1^Health and Social Care Economics Group, Caring Futures Institute, Flinders University, Australia

^2^Health Economics Division, School of Public Health and Preventive Medicine, Monash University, Melbourne, Victoria, Australia

**Corresponding Author**: Kiri Lay, Health and Social Care Economics Group, Caring Futures Institute, Flinders University, Adelaide, GPO Box 2100, Adelaide, SA 5001, Australia. E: [kiri.lay@flinders.edu.au](mailto:kiri.lay@flinders.edu.au)

Online Supplementary Information 1.

| **Comprehension -** Refers to understanding of words, content or item description – Issue where the question is understood differently to the way the instrument developers intend it to be understood or where words or content are not understood at all. | |
| --- | --- |
| Response Issue Recorded | *I have as much independence as I want. I suppose that’s none of the time, isn’t it? Because of the mobility* P131 |
| No Response Issue Recorded | *Independent? Well, I can’t, my independence, well I can’t do anything by myself really. Well, that’s not quite true is it, if I can make decisions. As much independence as I want. Well, I suppose I have as much independence as I want.* P117 |
| **Recall -** Refers to the timeframe considered and the ability to recollect necessary and relevant information – issues where recollected information is from the incorrect time period or there is an inability to recall information from the appropriate time frame. | |
| Response Issue Recorded | *I have as much independence as I want. It means I have much independence, but then sometimes in the household when we’re working together – my wife or I – on whatever we’re doing, sometimes it doesn’t blend together. She gets a bit cranky with me and vice versa*. P125 |
| No Response Issue Recorded | *Have as much independence as I want? I think that’s a fairly honest answer. You’ve only got to do what they ask you to do if it’s necessary and you’re allowed to do what pleases you otherwise. They’ve got activities on all the time pretty well.* P136 |
| **Judgment -** Refers to the process of weighing up the retrieved information – issues where under or overreporting of health status | |
| Response Issue Recorded | *I have leisure activities, hobbies I enjoy. Yes, all the time. If I could, that stone work that I do, I’d still do it, but I’ve got to understand it’s a bit of a messy job. I can’t – if they said to me “Oh, George, we want to take you to a place in [town], because we’ve got a block of [Town] stone there, and we want to you to explain how that becomes ashlars and then put into a house.” And it’s a big block of stone – it’s – what’d it be – four feet by three feet by three feet. And what you do, when they put that on the block, you cut it in half and that half you cut it into three slabs. And then you tick down one of them slabs, and you cut that into ashlars, and that’s what they build houses and things with.* P251 |
| No Response Issue Recorded | *I have leisure activities/hobbies I enjoy. Well, I haven't done much since I've been in here, but I have – If I could do some, I would be doing it. I suppose I've got to put that the answer to that is none of the time at the – Because, I can't do much at the moment. At the present moment I can't do – At the present moment, I have not – Oh god, got hobbies.* P129 |
|  |  |
|  |  |
|  |  |
|  |  |
| **Response mapping –** Refers to the formatting of the recalled information into the available response options – Issues include inappropriately applying response categories or responses OR stated answer (verbal protocol data) misaligned with chosen answer (survey response data). | |
| Response Issue Recorded | *No. I’m pretty lucky I don’t experience any pain When I experienced pain means – I don’t anyway. Well, I say – if that’s again I don’t experience any pain. Well, that’s really hard because I honestly don’t get any pain. No pain anywhere. As I said before I don’t have any pain. Not at all I don’t. No pain. Pain doesn’t worry me much anyway. I don’t have it anyway.* P205 |
| No Response Issue Recorded | *Most of the time they manage it. I get headaches, and then they give me a couple of Panadol –it fixes that.* P210 |
| **Struggle -** Participant needed assistance with task completion – including redirection to task and assistance with correct answering (i.e. selection of response) | |
| Response  Issue | Participant: *Okay. It says I am able to get around as much as I want to with the mobility of aids wheelchair, walker, stick, if I used on: all of the time; most of the time; some of the time; a little of the time; none of the time.*  Interviewer: *So, if you just deal with the first question first. So, you need to choose one of those responses: all of the time; some of the time; none of time.* Participant *Number three.*  Interviewer*: Which is, which one's that?*  Participant: *I generally have it* –*Do you want me to do number two?* Interviewer: *Sorry, I'll just pop it there, so I can see. So, you need to answer this first question, which is I am able to get around as much as I want to. And then, choose one of the options from: all of the time; most of the time; a little of the time; or none of the time.*  Participant: *Okay. If you use them, all of the time. Well, it'll be one*.  Interviewer: *Which is, which one's that, all of the time?*  Participant: *All of the time.* P128 |
| No Response Issue Recorded | *I can get around all the time. I can walk freely, but the two worn out knees is my biggest struggle, and I need a walker to steady me, so I’ll tick that.* P111 |
